# Preclinical Profile of the HIV-1 Maturation Inhibitor VH3739937

**DOI:** 10.3390/v16101508

**Published:** 2024-09-24

**Authors:** Brian McAuliffe, Paul Falk, Jie Chen, Yan Chen, Sing-Yuen Sit, Jacob Swidorski, Richard A. Hartz, Li Xu, Brian Venables, Ny Sin, Nicholas A. Meanwell, Alicia Regueiro-Ren, David Wensel, Umesh Hanumegowda, Mark Krystal

**Affiliations:** 1 ViiV Healthcare, 36 East Industrial Road, Branford, CT 06405, USA; brian.v.mcauliffe@viivhealthcare.com (B.M.); paul.j.falk@viivhealthcare.com (P.F.); david.l.wensel@viivhealthcare.com (D.W.); umesh.m.hanumegowda@viivhealthcare.com (U.H.); 2 Bristol Myers Squibb, 5 Research Parkway, Wallingford, CT 06492, USA; chenjiez@yahoo.com (J.C.); ylisachen@yahoo.com (Y.C.); sitsy@yahoo.com (S.-Y.S.); jacob.swidorski@bms.com (J.S.); rhartz712@yahoo.com (R.A.H.); li.xu1@bms.com (L.X.); brian.venables@bms.com (B.V.); ny.sin@ucsf.edu (N.S.); nicholas.meanwell@gmail.com (N.A.M.); alicia.regueiroren@bms.com (A.R.-R.)

**Keywords:** antiviral activity, diverse HIV-1 subtypes, dissociative half-life, resistance selection

## Abstract

The HIV-1 maturation inhibitor (MI) VH3739937 (VH-937) inhibits cleavage between capsid and spacer peptide 1 and exhibits an oral half-life in humans compatible with once-weekly dosing. Here, the antiviral properties of VH-937 are described. VH-937 exhibited potent antiviral activity against all HIV-1 laboratory strains, clinical isolates, and recombinant viruses examined, with half-maximal effective concentration (EC_50_) values ≤ 5.0 nM. In multiple-cycle assays, viruses less susceptible to other MIs, including A364V, were inhibited at EC_50_ values ≤ 8.0 nM and maximal percent inhibition (MPI) values ≥ 92%. However, VH-937 was less potent against A364V in single-cycle assays (EC_50_, 32.0 nM; MPI, 57%) and A364V emerged in one of four resistance selection cultures. Other substitutions were selected by VH-937, although re-engineered viruses with these sequences were non-functional in multiple-cycle assays. Measured dissociation rates from wild-type and A364V-containing VLPs help explain resistance to the A364V mutation. Overall, the in vitro antiviral activity of VH-937 supports its continued development as a treatment for HIV-1.

## 1. Introduction

Drug-resistant HIV-1 strains remain an obstacle in the pursuit of ending the AIDS epidemic. Recent studies have reported 7% to 19% of transmitted HIV-1 strains have ≥1 antiretroviral drug resistance mutation [1,2,3], 45% to 82% of people using antiretroviral therapy (ART) have viruses resistant to ≥1 inhibitor class [3,4,5], and 9% to 15% of people with prior ART experience harbor multidrug-resistant HIV-1 with reduced susceptibility to ≥3 classes of antiretroviral agents [4,5]. As multidrug resistance can prevent the construction of suppressive antiretroviral regimens for people living with HIV-1, inhibitors with mechanisms of action distinct from existing antiretroviral classes are needed. Ideally, these inhibitors will have broad antiviral activity across HIV-1 subtypes and demonstrate no cross-resistance to other antiretroviral agents. In addition, new agents would preferably have long-acting potential, which provide people living with HIV-1 high treatment satisfaction and improve treatment adherence [6,7].

The maturation step of the HIV-1 life cycle presents a promising target for novel therapeutics [8]. During maturation, structural changes take place inside HIV-1 virions in tandem with cleavage of the structural polyprotein Gag by the HIV-1 protease. This series of events ultimately results in the formation of a ribonucleoprotein core necessary for completing the post-entry steps of the viral life cycle [8]. Notably, altering the efficiency of any of the steps in the maturation process can greatly diminish viral infectivity, though certain steps in particular are more sensitive to inhibition [9,10,11]. This strategy of selectively blocking a specific step of HIV-1 maturation is distinct from that of HIV-1 protease inhibitors and represents the mechanism of action underpinning the HIV-1 maturation inhibitor (MI) class. More specifically, HIV-1 MIs interfere with the final step in viral maturation by blocking the removal of spacer peptide 1 (SP1) from the C-terminal end of capsid (CA) [12]. Proof-of-concept for MIs as antiretroviral agents was initially provided by bevirimat. In phase 1 clinical trials, bevirimat demonstrated a favorable safety and efficacy profile [13]. In phase 2 clinical trials that enrolled adults living with HIV-1 and CD4+ T-cell counts >200 cells/mm^3^ who were naive to ART or temporarily not using ART, bevirimat exhibited efficacy in only a subset of subjects due to a high frequency of naturally occurring polymorphisms associated with reduced susceptibility to bevirimat [13,14]. Consequently, its clinical development was ultimately discontinued [14]. Each subsequent investigational MI, such as GSK2838232, GSK3532795 (BMS-955176), and GSK3640254, extended the range of viral sequences susceptible to inhibition while also optimizing the safety and tolerability profile of MIs [12,15,16,17]. GSK3640254 demonstrated robust antiviral activity against a range of HIV-1 isolates with diverse Gag sequences, including many with polymorphisms or substitutions that had reduced sensitivity to other MIs, though the A364V substitution remained less susceptible to inhibition [12]. The reduced susceptibility of the A364V mutation is thought to be associated with its relatively rapid cleavage of p25 by HIV-1 protease, substantially reduced MI residence times, and poorer affinity to previous- and current-generation MIs [12]. In the phase 2b DOMINO and DYNAMIC clinical trials, participants naive to ART administered GSK3640254 in combination with dolutegravir or two nucleoside reverse-transcriptase inhibitors demonstrated generally comparable efficacy, safety, and tolerability to dolutegravir-based two- or three-drug regimens, with no treatment-emergent resistance observed through 24 weeks [18,19].

VH3739937 (VH-937; previously GSK3739937) is another investigational MI and is structurally similar to GSK3640254. Like GSK3640254, VH-937 interferes with capsid/spacer peptide 1 cleavage, has a low nanomolar potency in vitro, and exhibits significantly improved pan-genotypic coverage and potency against Gag polymorphisms relative to prior MIs [20]. In a phase 1 study in HIV-negative adults, VH-937 administration was generally well tolerated under short-term administration and caused no unexpected safety concerns after single or multiple doses [20]. Although earlier MIs had half-lives consistent with daily dosing, VH-937 has a longer oral half-life of 67 to 97 h [20]. Thus, unlike other developmental HIV-1 MIs, VH-937 is predicted to have a dosing schedule that is less frequent than once daily [20]. Here, we report the in vitro potency of VH-937 against a broad range of HIV-1 sequences, identify substitutions associated with reduced susceptibility, and confirm its mechanism of action.

## 2. Materials and Methods

### 2.1. Cells, Compounds, and Viruses

**Figure 1:**
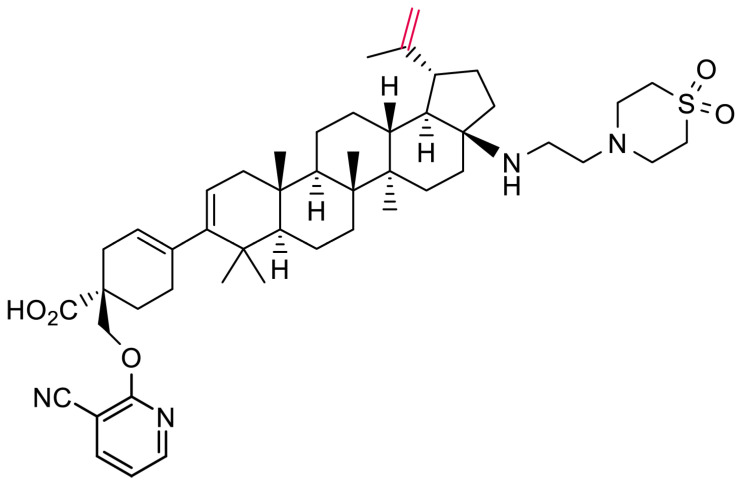
Chemical structure of VH3739937. The C20/29 double bond (red) was tritiated to create ^3^[H]-BMT-266754, the radiolabeled surrogate for VH3739937.

MT-2, CEM-NKR-CCR5-Luc, and PM1 cells and HIV clinical isolates were acquired from the National Institutes of Health (NIH) AIDS Research and Reference Reagent Program and HEK 293T cells from the American Type Culture Collection (ATCC, BEI Resources, Manassas, VA, USA). B6 cells, originating from the DuPont Pharmaceutical Company (Wilmington, DE, USA), are an MT-4 cell line containing an integrated firefly luciferase gene under the control of the HIV long-terminal repeat [15]. Peripheral blood mononuclear cells (PBMCs) were obtained by density gradient centrifugation of whole-blood samples collected from healthy donors through the Gulf Coast Regional Blood Center (Houston, TX, USA). The cells were cultured as previously described [12]. CEM-SS cells were obtained through the NIH AIDS Research and Reference Reagent Program. MDCK canine kidney cells, HEp2 human epithelial HeLa derivative cells, and H1-HeLa cells were obtained from ATCC. Huh7 cells were obtained from Dr. Ralf Bartenschlager (Department of Molecular Virology, Hygiene Institute, University of Heidelberg, Heidelberg, Germany) by ImQuest BioSciences, Inc. (Frederick, MD, USA) through a specific licensing agreement [21]. The tetracycline-inducible HepG2-AD38 cell line was gifted from Phil Furman at Pharmasett (Princeton, NJ, USA) [22]. VH-937 was prepared at ViiV Healthcare, atazanavir was prepared at Bristol Myers Squibb, and nelfinavir (NFV) was purchased from commercial sources. ^3^[H]-BMT-266754, the radiolabeled surrogate of VH-937, was synthesized at Bristol Myers Squibb by tritiating the C20/C29 double bond of VH-937 (Figure 1).

Laboratory-adapted HIV-1 and HIV-2 strains were obtained from the NIH AIDS Research and Reference Reagent Program and propagated in MT-2 or PM1 cells. NLRepRlucP373S was derived from NL_4-3_. Initially, NL_4-3_ was modified to replace a portion of *nef* with the *Renilla* luciferase gene, creating NLRepRluc [23]. NLRepRluc was then further modified to include a substitution in spacer peptide 2 of Gag (P373S) to reflect the predominant HIV-1 subtype B sequence. Site-directed mutant (SDM) versions of NLRepRlucP373S or NL_4-3_ were created to harbor substitutions in Gag associated with reduced susceptibility to previous investigational MIs and substitutions identified during dose-escalating resistance selection with VH-937. A version of NLRepRlucP373S in which *env* was deleted (NLRepRlucP373SΔenv) was also generated for use in single-cycle pseudotyping assays. Gag sequences from a panel of clinical isolates representing diverse HIV-1 subtypes were obtained from the NIH AIDS Research and Reference Reagent Program or Bristol Myers Squibb (informed consent was obtained) and inserted into the NLRepRlucP373S proviral clone [12]. Recombinant virus stocks were produced by transfection of HEK 293T cells (Lipofectamine™ LTX Reagent with PLUS™ Reagent kit; Invitrogen, Waltham, MA, USA) and expansion in MT-2 cells. Titers for recombinant virus stocks were determined by infection of MT-2 cells under standard conditions with serial dilutions of the virus stock and monitoring for *Renilla* luciferase activity. Clinical isolates were grown through infection and expansion in phytohemagglutinin-activated CD8+ T-cell-depleted PBMCs and monitored for reverse-transcriptase activity, as described below.

### 2.2. Drug Susceptibility Assays

#### 2.2.1. Multiple-Cycle Replication Assays

Susceptibility was measured using MT-2 cells for viruses containing the *Renilla* luciferase gene, CEM-NKR-CCR5-Luc or B6 cells for reporter-free wild-type and SDM laboratory strains, and CEM-NKR-CCR5-Luc cells or PBMCs for clinical isolates, similarly to a previously published procedure [12]. For MT-2 and CEM-NKR-CCR5-Luc cell assays, the cells were seeded in 384-well plates at a density of 9.5 × 10^3^ cells/well and infected at a multiplicity of infection of 0.005 to 0.01 in the presence of 3-fold serially diluted VH-937 (starting concentration of 10 µM) at a final dimethyl sulfoxide (DMSO) concentration of 1%. After 3 to 4 days (reporter viruses) or 5 to 6 days (reporter-free viruses incubated with CEM-NKR-CCR5-Luc cells), virus growth was measured by the amount of expressed luciferase, which was quantified using the EnduRen™ Live Cell substrate (Promega, Madison, WI, USA). The growth of clinical isolates in PBMCs was quantified on day 7 postinfection by reverse-transcriptase activity in a scintillation proximity assay using a TopCount (Packard Bioscience, Meriden, CT, USA) luminometer. The half-maximal effective concentration (EC_50_) for all assays was calculated using the exponential form of the median effect equation where percent inhibition = 100 × {1/[1 + (EC_50_/drug concentration)*^m^*]}, where *m* is a parameter that reflects the slope of the concentration–response curve. Maximal percent inhibition (MPI) values were calculated as MPI = 100 − (mean of the signals at the 2 highest concentrations of compound/mean signal of 2 no-drug control wells) × 100.

#### 2.2.2. Single-Cycle Replication Assays

Single-cycle assays were performed similarly to a previously described procedure [15]. Briefly, 1.5 µg of a plasmid containing the coding sequence for NLRepRlucP373SΔenv and 1.5 µg of a plasmid carrying the sequence for the murine leukemia virus envelope SV-A-MLV-env (acquired from the NIH AIDS Research and Reference Reagent Program) were co-transfected into HEK 293T cells in the presence of either VH-937, NFV (protease inhibitor control), or an equivalent amount of DMSO (no-drug control). After overnight incubation, the transfected cells were co-seeded with fresh (non-transfected) HEK 293T cells at a 1:5 ratio and a density of 9.5 × 10^3^ cells/well in a new 384-well plate. After 72 h of incubation, cell-associated luciferase activity was measured using the EnduRen™ Live Cell substrate as above. Single-cycle EC_50_ values were calculated as the compound concentration that inhibits 50% of the maximal signal produced by the no-drug control (correcting for background). Background was determined as the residual signal observed upon inhibition at the highest NFV concentration (3000 nM). Half-maximal fold change effective concentration (FC-EC_50_) values were calculated by dividing the EC_50_ of recombinant or SDM viruses by the EC_50_ of the wild-type NLRepRlucP373SΔenv virus run in parallel.

#### 2.2.3. Cytotoxicity of VH-937

Cytotoxicity was assessed in MT-2 cells after a 4-day incubation in the presence of 5-fold serial dilutions of VH-937 (starting concentration, 50 μM), per published protocol [12,24]. Before the experiment was conducted, MT-2 cells were seeded at a density of 2.25 × 10^4^ cells/well in a 96-well plate. Half-maximal cytotoxic concentration (CC_50_) values were calculated by fitting the data to a 4-parameter logistic formula, y = A + {[B − A]/[1 + ([C/x]^D)]}, where A and B denote minimal percent inhibition and MPI, respectively, C is the half-maximal inhibitory concentration, D is the Hill slope, and x represents VH-937 concentrations.

Cytotoxicity was also evaluated in CEM-SS, MDCK, HEp2, H1-HeLa, Huh7, and HepG2 cells after incubation with half-log serial dilutions of VH-937 (starting concentration, 20 µM). The cells were added to 96-well microtiter plates, and the cytotoxicity for each cell type was evaluated in duplicate wells per concentration. CEM-SS cells were seeded at a density of 2.5 × 10^3^ cells/well in RPMl1640 medium (Gibco 32404-014) supplemented with 10% heat-inactivated fetal bovine serum (FBS), 2 mM of L-glutamine (Lonza 17-605E), and 100 U/mL of penicillin/100 μg/mL of streptomycin (Lonza 17-602E) and incubated for 6 days. MDCK cells were seeded at a density of 1 × 10^4^ cells/well in Dulbecco’s Modified Eagle Medium (DMEM) supplemented with 10% FBS, 2 mM of L-glutamine, 100 U/mL of penicillin/100 µg/mL of streptomycin, 1 mM of sodium pyruvate (Lonza 13-115E), and 0.1 mM of non-essential amino acids (Lonza 13-114E) and incubated for 4 days. HEp2 cells were seeded at a density of 5 × 10^3^ cells/well in DMEM supplemented with 10% FBS, 2 mM of L-glutamine, and 100 U/mL of penicillin/100 µg/mL of streptomycin and incubated for 6 days. H1-HeLa cells were seeded at a density of 5 × 10^3^ cells/well in DMEM supplemented with 10% FBS, 2 mM of L-glutamine, 100 U/mL of penicillin, and 100 µg/mL of streptomycin and incubated for 6 days. Huh7 cells were seeded at a density of 5 × 10^3^ cells/well in DMEM (Gibco 31053-028) supplemented with 10% heat-inactivated FBS (Gibco 16140-089), 2 mM of L-glutamine, 100 U/mL of penicillin/100 ug/mL of streptomycin, and 0.1 mM of non-essential amino acids plus 1 mg/mL of G418 (VWR 091672548) and incubated for 72 h. The tetracycline-inducible HepG2-AD38 cell line was seeded at a density of 5 × 10^4^ cells/well in DMEM: Nutrient Mixture F-12 (Gibco 11320033) supplemented with 10% heat-inactivated FBS, 50 µg/mL of penicillin and 50 µg/mL of streptomycin, 100 µg/mL of kanamycin (Sigma K0254), 0.3 µg/mL of tetracycline (Sigma T7660), and 400 µg/mL of G418 and incubated for 7 days.

Following the incubation periods, uninfected cell monolayers were stained with XTT-tetrazolium dye (Thermo Fisher Scientific, Waltham, MA, USA), and data were collected spectrophotometrically at 450 and 650 nM using Softmax 5.4.2 software (Molecular Devices, San Jose, CA, USA). The CC_50_ was evaluated against untreated control cells.

#### 2.2.4. Effect of Human Serum

The effect of human serum on the antiviral activity of VH-937 toward NLRepRlucP373S virus was determined using a drug sensitivity assay in MT-2 cells seeded in 96-well plates at a density of 2.25 × 10^4^ cells/well with standard assay medium (RPMI 1640, 10% heat-inactivated fetal bovine serum, 100 U/mL of penicillin G, 100 µg/mL of streptomycin, 2 mM of L-glutamine, 10 mM of HEPES, pH 7.55; all reagents from Gibco^®^, Gaithersburg, MD, USA) supplemented with 40% human serum (BioReclaimation, cat.# HMSRM-HI) and an additional 27 mg/mL of human serum albumin (HSA, Sigma-Aldrich, St. Louis, MO, USA) to yield a final HSA concentration of approximately 45 mg/mL. This final concentration approximates the concentration of albumin in 100% human serum [25]. Luciferase activity was used as the endpoint.

### 2.3. Resistance Selection Assays

MT-2 cells (1 × 10^6^) in T25 flasks or 24-well plates were infected with a full-length HIV-1 NL_4-3_ or IIIB virus at a multiplicity of infection of 0.005. Sixteen hours postinfection, the cells were treated with 1.8 nM of VH-937 (approximately 1 × EC_50_) or an equal volume of DMSO (final concentration, 0.2%). The cell cultures were visually monitored every 1 to 3 days for virus-induced cytopathic effects (CPE). In the absence of appreciable CPE, the cell cultures were refreshed every 3 to 4 days by removing approximately half of the cells and medium and adding an equal volume of fresh growth medium while maintaining the same inhibitor or DMSO concentration. When CPE were apparent, culture supernatant with double the concentration of VH-937 was used to infect fresh MT-2 cells. For passage, the cells were seeded at a density of 2 × 10^5^ cells/mL in a T25 flask (10 mL) or 24-well plate (3 mL). At each passage, the cells were pelleted and frozen at −80 °C. After 7 passages, the experiment was terminated and samples from that passage were used for genotypic analyses of Gag.

For the genotypic analysis of Gag, total cellular DNA was extracted from each cell pellet using the DNeasy Blood & Tissue Kit (QIAGEN, Hilden, Germany) according to manufacturer’s protocol. Amplicons containing the *gag*/*pro* coding region were obtained through polymerase chain reaction by using the forward primer TCTCTCGACGCAGGACTCGGCTTGCTG and reverse primer CCAATTCCCCCTATCATTTTTGGTTTCCAT. Purified amplicons were submitted for population sequence analysis by GENEWIZ and analyzed using Lasergene (DNASTAR, Madison, WI, USA). The frequency of Gag variants was estimated from sequence traces of polymerase chain reaction amplicons and protein alignment.

### 2.4. Preparation of HIV-1 Virus-like Particles

Noninfectious virus-like particles (VLPs) were produced as previously published [15]. Briefly, HEK 293T cells at 70% to 80% confluency in a T175 flask were transfected with 18 µg of a plasmid containing the codon-optimized coding sequence for the full-length HIV-1 LAI Gag polyprotein under the control of the cytomegalovirus promoter using the TransIT-LT1 reagent (Mirus Bio LLC, cat# MIR2300, Madison, WI, USA) or with plasmids coding for mutant sequences containing substitutions of interest. After 2 days, supernatants containing secreted VLPs were cleared from cell debris by filtration (0.45 µm filter, Millipore cat# SCHVU01RE), pelleted through a 20% sucrose cushion in phosphate-buffered saline (25,000 rpm for 2 h), re-suspended in phosphate-buffered saline at a total protein concentration of 1000 µg/mL, and stored at −80 °C. Total protein concentration was measured using a Bradford assay.

### 2.5. VLP Cleavage Assay

As previously described [26], cleavage between CA and SP1 was evaluated via proteolysis of VLPs. In short, ~100 ng of delipidated VLPs were pre-incubated with 3 µM of VH-937 or an equivalent amount of DMSO (final concentration, 0.1%) at 22 °C for 2 h and then mixed with 0.27 µM of an HIV-1 protease resistant to auto-proteolysis [27]. Aliquots were removed 0, 0.5, 2, and 4 h after mixing and digested overnight with trypsin at 37 °C. The samples were then analyzed by liquid chromatography with mass spectrometry (LCMS) using a nanoACQUITY UPLC^®^ System (Waters Corporation, Milford, MA, USA) interfaced with an LTQ XL™ Orbitrap mass spectrometer (Thermo Fisher Scientific, Waltham, MA, USA). The data were acquired using an Advance CaptiveSpray ion source (Michrom Bioresources Inc., Auburn, CA, USA).

### 2.6. Specific Binding and the Kinetics of Dissociation from HIV-1 Gag VLPs

Dissociative half-lives of VH-937 to VLPs were determined using the radiolabeled VH-937-surrogate compound ^3^[H]-BMT-266754 in a scintillation proximity assay (SPA). Virus-like particles were mixed with 20 nM of ^3^[H]-BMT-266754 for 3 h at room temperature in 40 µL of PBS with SPA beads (100 µg/well; PVT WGA SPA beads, PerkinElmer cat# RPNQ0250) and allowed to equilibrate, followed by the addition of >50-fold excess VH-937 at room temperature. At different time points, bound ^3^[H]-BMT-266754 was measured using a Top Count or Microbeta2 plate reader (PerkinElmer, Waltham, MA, USA), and the data were analyzed via GraphPad Prism software (version 5.1).

## 3. Results

### 3.1. Antiviral Activity, Cytotoxicity, and Estimated Minimum Clinically Effective Plasma Concentration of VH-937

The antiviral activity of VH-937 (Figure 1) against the reporter virus NLRepRlucP373S was evaluated in a multiple-cycle replication assay with MT-2 cells. The mean (SD) EC_50_ determined from 23 independent experiments was 1.8 (1.1) nM, and the mean (SD) 90% effective concentration (EC_90_) was 12.9 (9.4) nM (Table 1).

The cytotoxicity of VH-937 in MT-2 cells was investigated, and the mean (SD) CC_50_ was 11.4 (5.0) µM. The cytotoxicity of VH-937 was also investigated in CEM-SS, MDCK, HEp2, H1-HeLa, Huh7, and HepG2 cell lines, and VH-937 CC_50_ values in these cell lines were 4.19, 0.54, 0.36, 0.37, 10.1, and 11.4 µM, respectively. Together, these results suggest VH-937 is a highly potent and selective inhibitor of the NLRepRlucP373S reporter virus.

The effect of human serum on the activity of VH-937 was determined by comparison of EC_50_ values derived from the multiple-cycle assay performed under standard conditions (i.e., 10% fetal bovine serum) and in medium supplemented with human serum (40% human serum + 27 mg/mL of HSA). Under high-serum conditions, the mean (SD) EC_50_ was 26.0 (3.3) nM, yielding a serum shift factor of 14.4-fold and giving an implied protein-bound fraction of 93.1% in 100% equivalent human serum. Based on these data and to establish a target minimum plasma concentration for clinical efficacy, the EC_90_ and three times the protein-binding-adjusted EC_90_ values were determined for a panel of six viruses with different Gag phenotypes. These viruses were chosen based on studies with earlier MIs and represent a selection of sequences observed in the virus population with a range of susceptibilities to MIs. VH-937 potently inhibited all six viruses under standard conditions with EC_90_ values ≤ 10.0 nM. Using the mean value of three times the protein-binding-adjusted EC_90_ (Table 2), a target trough value of 312 nM was established for clinical use of VH-937.

### 3.2. Antiviral Activity against Laboratory Strains

VH-937 antiviral activity was evaluated against a panel of eight HIV-1 laboratory strains and two HIV-2 strains in CEM-NKR-CCR5-Luc cells. VH-937 was highly potent against the HIV-1 strains, with EC_50_ values ranging from 1.3 to 4.4 nM (Table 3).

The mean (SD) EC_50_ was 2.2 (0.3) nM for CCR5-tropic viruses and 2.3 (1.2) nM for CXCR4-tropic viruses. VH-937 was active against the ROD HIV-2 strain (mean [SD] EC_50_, 1.3 [0.2] nM) at a similar potency as the HIV-1 strains but was not active against the HIV-2 strain 287. Excluding this HIV-2 strain, the overall potency range for VH-937 was similar to that observed for atazanavir in side-by-side experiments.

### 3.3. VH-937 Robustly Inhibits HIV-1 Clinical Isolates of Different Subtypes

The antiviral activity of VH-937 was evaluated against a panel of 25 clinical isolates in freshly prepared PBMCs using reverse-transcriptase activity as an endpoint. VH-937 was highly potent against all 25 viruses, with EC_50_ values ranging from 1.0 to 5.0 nM (Table 4).

No discernible difference was apparent in the susceptibility of viruses from different HIV-1 subtypes to VH-937, with subtypes A, CRF01_AE, B, C, D, and F represented. In an alternative approach, a series of 16 different clinical isolates was used to directly infect CEM-NKR-CCR5-Luc cells. Again, high potency was observed for all 16 viruses, with EC_50_ values ranging from <1.0 to 4.0 nM. Additionally, HIV-1 isolates from type N and type O were tested and both were highly susceptible, with EC_50_ values of 3.1 and 4.7 nM, respectively. In total, all 41 HIV-1 clinical isolates examined were susceptible to VH-937 at EC_50_ values ≤ 5.0 nM, and no discernible difference in susceptibility was evident among the six HIV-1 subtypes examined. These data suggest VH-937 should maintain high potency against diverse clinical isolates.

### 3.4. Antiviral Activity of VH-937 against Polymorphic Viruses on an NLRepRlucP373S Background

Studies on earlier MIs have identified polymorphisms and substitutions that can affect the susceptibility of HIV-1 strains to certain MIs. To probe whether these variants also exhibit reduced susceptibility to VH-937, SDM viruses were created on the NLRepRlucP373S background and assessed. All SDM viruses examined in the multiple-cycle assay were potently inhibited by VH-937, with FC-EC_50_ values between 1.0 and 4.0 and MPI values ≥92% (Table 5). No undesired mutations on the HIV-1 open reading frames were observed following SDM.

Notably, the A364V mutant (previously shown to be selected for resistance to MIs) [12,28,29,30,31,32,33] had an EC_50_ of 5.0 nM and an MPI of 95%, which represented only a 2.5-fold change in VH-937 EC_50_ relative to the wild-type NLRepRlucP373S virus. Thus, in a multiple-cycle assay, VH-937 remained active against a virus harboring the A364V substitution.

In the single-cycle assay, VH-937 fully inhibited most of the panel of Gag polymorph–harboring substitutions that affect its susceptibility to previous MIs. For viruses including polymorphisms at positions 362, 370, and/or 371, the FC-EC_50_ compared with wild-type viruses ranged from 1.2 to 4.8 and the MPIs from 96% to 100% (Table 5). However, the A364V substitution caused a 6.4-fold increase in VH-937 EC_50_ (32.0 nM) and lowered the MPI to 57%. Another SDM virus harboring an A366V substitution also exhibited reduced susceptibility to VH-937, although it had a negative MPI value (−141%) indicative of drug-dependent growth. Altogether, most viruses with polymorphisms known to impact susceptibility to MIs remained sensitive to VH-937 in both multiple- and single-cycle assay formats. However, the decreased MPI observed with A364V in the single-cycle assay suggests there could be breakthrough of this virus in the presence of VH-937.

### 3.5. Selection of Viruses with Reduced Susceptibility to VH-937

To select for viruses with reduced susceptibility to VH-937, MT-2 cells were infected with either NL_4-3_ or IIIB in the presence of a sub-optimal concentration of VH-937 (1 × EC_50_) to allow low-level virus growth. Culture supernatants were grown until CPE appeared and were used to infect new cells in the presence of twice the previous VH-937 concentration such that the concentration of VH-937 in the final passage was 64 times the EC_50_. After outgrowth at the final VH-937 concentration, viral DNA was isolated, the Gag gene was sequenced, and the sequences were examined for differences from wild-type NL_4-3_ or IIIB (Table 6).

A364V was selected under escalating VH-937 conditions in one of the four selection assays but had not become fixed before termination of the experiment. Other substitutions that were selected included L363W, the double mutant D93N/T332P, and the triple mutant H144Y/V362I/R384K. Additionally, a mixture of amino acids was observed at position 298 (D298D/N) in the H144Y/V362I/R384K culture.

Each substitution identified during resistance selection was introduced into NL_4-3_-based viruses as individual or aggregate changes and tested for susceptibility to VH-937. In a multiple-cycle assay, only 5 (D93N, H144Y, V362I, A364V, and R384K) of the 10 mutant viruses grew enough to enable the analysis of effective concentrations, and none exhibited an FC-EC_50_ > 2.5 (Table 7).

A low MPI of 75% was observed for H144Y, but whether this reflected a reduced susceptibility to VH-937 was unclear because the NFV control sample similarly had a low MPI of ~74%. In a single-cycle assay, only D298N did not produce a measurable signal. Viruses including individual D93N, H144Y, or R384K substitutions exhibited EC_50_ values within 4.2-fold that of the wild type and MPIs ≥ 94%. Additionally, both the T332P-containing viruses and L363W-containing viruses exhibited high-level resistance to VH-937 in the single-cycle assay, with EC_50_ values > 2000 nM and very low MPIs. Thus, the dose-escalating resistance selection identified T332, L363, and A364 as key positions of interest due to their impact on virus susceptibility to VH-937.

### 3.6. VH-937 Mechanism of Action

**Figure 2:**
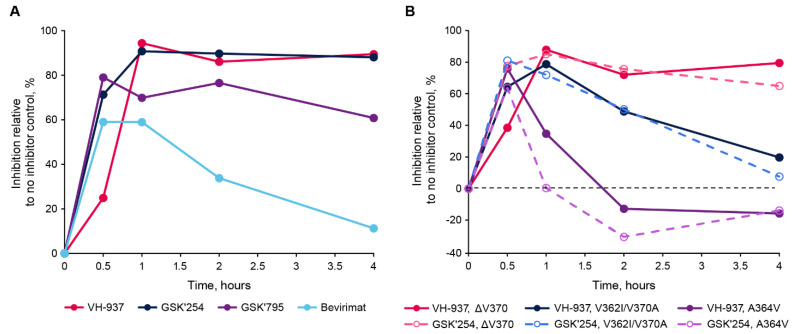
Inhibition of p24 appearance in a virus-like particle cleavage assay. (**A**) Delipidated wild-type or (**B**) mutant virus-like particles were pre-incubated with VH3739937, GSK3640254, GSK3532795, bevirimat, or dimethyl sulfoxide and then mixed with HIV-1 protease. Aliquots removed at the indicated time points were digested overnight with trypsin and then analyzed by liquid chromatography with mass spectrometry. Percent inhibition was measured relative to the appearance of p24 in the dimethyl sulfoxide control. GSK’254, GSK3640254; GSK’795, GSK3532795; VH-937, VH3739937.

The ability of VH-937 to inhibit HIV-1 replication by interfering with cleavage of p25 into CA and SP1 was confirmed using a VLP-based assay. The ability of VH-937 to block the cleavage of p25 from VLPs containing ΔV370, V362I/V370A, and A364V substitutions was examined. VH-937 demonstrated high-level inhibition of p25 cleavage without loss over time against the wild-type and ΔV370 VLPs (Figure 2). However, the inhibition of p25 cleavage with the V362I/V370A and A364V VLPs decreased with time, suggesting VH-937 may dissociate faster from these partially cleaved VLPs over time.

To examine this possibility, the dissociative half-lives of VH-937 for the wild-type, V362I/V370A, and A364V VLPs were determined. The dissociation of VH-937 from the VLPs was assessed by displacement of the radiolabeled compound with an excess of the unlabeled compound. Under the conditions used, the estimated dissociative half-life of VH-937 to wild-type VLPs was ~3 days (4125 min), indicating the compound dissociates relatively slowly. VH-937 also dissociated relatively slowly (albeit slightly faster than from the wild type) from V362I/V370A VLPs (dissociative half-life, 2894 min). However, the dissociative half-life with A364V-containing VLPs was relatively rapid at 29 min. Altogether, these data suggest VH-937 readily binds to VLPs, including those harboring A364V, and that the potentially reduced susceptibility of A364V-containing viruses is due to a faster rate of dissociation.

## 4. Discussion

VH-937 is an HIV-1 MI with an oral half-life of ~3 days in healthy participants without HIV, a time frame that exceeds all prior investigational HIV-1 MIs [20]. This includes the related GSK3532795 and GSK3640254, each of which demonstrated virologic efficacy in phase 2b clinical trials in people living with HIV-1 who are naive to treatment with a viral load ≥ 1000 copies/mL and CD4+ T-cell counts ≥ 200 cells/mm^3^ [18,19,30]. In this study, the in vitro virologic profile of VH-937 was evaluated. Against all 8 HIV-1 laboratory strains and all 41 clinical isolates from the various subtypes examined, VH-937 exhibited low nanomolar potency, thereby demonstrating that it has pan-genotypic coverage of HIV-1 subtypes. Viruses with a range of Gag polymorphisms associated with resistance to prior MIs, including A364V, were also highly susceptible to VH-937 in multiple-cycle assays, with EC_50_ values between 2.0 and 8.0 nM and MPI values ≥ 92%. However, VH-937 had a lower MPI in a single-cycle assay against HIV-1-harboring A364V and dissociated relatively rapidly from A364V-containing VLPs, suggesting viruses with the A364V mutation could still replicate to an extent in the presence of VH-937. Consistent with this possibility, A364V emerged in one of four cultures undergoing dose-escalating resistance selection. Nevertheless, these findings demonstrate the robust antiviral properties of VH-937 against HIV-1 strains with diverse Gag sequences and polymorphisms conferring resistance to prior MIs and support the continued clinical development of VH-937.

The A364V substitution has commonly appeared during resistance selection experiments with MIs as well as in phase 2 clinical trials with GSK2838232 and GSK3532795 [12,28,29,30,31,32,33]. Based on structural studies with bevirimat, MIs bind within the central channel of a six-helix bundle formed by hexamers of CA-SP1 proteins [34,35,36]. This “tightens” the bundle and stabilizes the CA-SP1 junction in a conformation the HIV-1 protease cannot recognize [34,37]. In this structure, A364 does not make direct contact with the MI but rather faces adjacent CA-SP1 molecules [35,36]. A364V reduces how tightly the helices can pack [34,35], and as a result, the interaction between bevirimat and the CA-SP1 region is left highly unstable or may even be completely prevented [34]. However, despite the potential lack of interaction with bevirimat, the partial activity observed with VH-937 against A364V-containing viruses indicates the substitution does not completely impede VH-937 from binding. Under the conditions used, the 29 min dissociative half-life for VH-937 from A364V-containing VLPs further suggests the A364V substitution destabilizes the interaction between VH-937 and CA-SP1 hexamers. Comparatively, GSK3532795 and GSK3640254 had ≤1 min dissociative half-lives from A364V-containing viruses [12], ostensibly explaining the improved antiviral potency VH-937 has against A364V-containing viruses in multiple- and single-cycle replication assays. Nevertheless, the resistance selection experiments demonstrated that the possibility for breakthrough of A364V-containing viruses in the presence of VH-937 remains.

Other single amino acid substitutions that reduced susceptibility to VH-937 included A366V, T332P, and L363W, though notably, A366V did not emerge during resistance selection, and T332P and L363W were non-functional in multiple-cycle replication assays. A366V has been identified as a compound-dependent resistance mutation with other MIs [12,29,38], and drug dependency was also observed with VH-937. Virus particle production with A366V is impaired [39] such that stabilization of the CA-SP1 lattice by MIs likely restores the efficiency with which virions assemble. Substitutions at position T332 have been reported during resistance selection for GSK3532795 (T332S) and GSK3640254 (T332P) [12,31], and, where examined, the drug dependency of those strains varied [12], suggesting substitutions at position T332 may not be sufficient to cause compound dependence. As position 363 is part of the MI binding interface [35,36], L363W alters direct contacts between the CA-SP1 six-helix bundle and VH-937. Since Trp is a much bulkier amino acid than Leu, L363W may sterically block VH-937 from accessing the binding site entirely. Notably, neither of the single amino acid substitutions that emerged during resistance selection are common polymorphisms, with just 0.03% of sequences in the Los Alamos National Laboratory HIV Database containing T332P and no instances of L363W [40]. This suggests these substitutions may be difficult to select for in vivo, as viruses would require compensatory substitutions to improve viral fitness.

In addition to the above individual amino acid substitutions, the triple mutant H144Y/V362I/R384K also conferred resistance to VH-937, although it was also non-functional in the multiple-cycle replication assay, and each individual substitution had a very limited effect. For V362I, this was consistent with prior observations that determined it worked synergistically with other changes to limit GSK3532795 potency [31]. Interestingly, both substitutions that partnered with V362I in this study were not located near the MI binding site. One of those substitutions was H144Y, which is part of an important β-hairpin structure in the N-terminal domain of CA that forms after cleavage between matrix and CA [8,41]. Conceivably, altering the β-hairpin could propagate changes through the remainder of CA that ultimately impact VH-937 binding [42]. The other substitution, R384K, is located within the nucleocapsid protein. How R384K might impact susceptibility to VH-937 is unclear, as nucleocapsid is separated from the C-terminal end of SP1 in the first step of HIV-1 maturation, long before cleavage between CA and SP1 is thought to take place [8]. Additionally, the experiment that selected for a H144Y/V362I/R384K-containing virus culture included a D298N substitution, although D298N was a mixture at the time the assay was halted. As an individual substitution, D298N was non-functional in both single-cycle and multiple-cycle assays. The location of D298N within the highly conserved major homology region (MHR) of CA may explain the reason for its lack of activity in the absence of other compensatory substitutions, as alterations to the MHR are highly detrimental to virion assembly [43]. Nevertheless, changes to the MHR have been associated with reduced potency of the MI PF-46396 [29], suggesting alterations in the MHR could provide a potential resistance pathway for MIs. The MHR includes a key contact point for the host co-factor inositol phosphate IP_6_, and IP_6_ has a similar effect of stabilizing the CA-SP1 lattice [8,44]. Thus, a resistance pathway to MIs might be via substitutions within the MHR that offset the stability MIs provide by altering interactions with IP_6_ [44].

A limitation of this study is that although VH-937 was tested against a broad range of HIV-1 sequences, this may only represent a subset of HIV-1 sequence variations. Additionally, the binding and dissociation experiments used surrogate molecules for VH-937. Even though these surrogates are close in structure and inhibitory activity to VH-937, there could be some differences in their profiles.

In this study, VH-937 exhibited low nanomolar potency against an extensive array of viruses from different HIV-1 subtypes containing diverse Gag polymorphisms and substitutions associated with resistance to other MIs. Additionally, VH-937 demonstrated better potency and MPI against viruses harboring the known resistance-associated substitution A364V compared with earlier investigational MIs. Overall, and in conjunction with the results of a phase 1 study demonstrating no unexpected safety or tolerability issues and an oral half-life of ~3 days in HIV-negative adults [20], the data presented here support the continued clinical development of VH-937 for the treatment of HIV-1.

## Figures and Tables

**Table 1:** Antiviral activity of VH3739937 against HIV-1 NLRepRlucP373S and cytotoxicity in MT-2 cells.

	VH3739937	N
Antiviral activity, standard conditions ^a^		
EC_50_, mean (SD), nM	1.8 (1.1)	23
EC_90_, mean (SD), nM	12.9 (9.4)	23
Cytotoxicity		
CC_50_, mean (SD), µM	11.4 (5.0)	16
Therapeutic index ^b^	6333	
Antiviral activity, high-serum conditions ^c^		
EC_50_, mean (SD), nM	26.0 (3.3)	15
Serum shift factor ^d^	14.4	

CC_50_, half-maximal cytotoxic concentration; EC_50_, half-maximal effective concentration; EC_90_, 90% effective concentration; FBS, fetal bovine serum; HS, human serum; HSA, human serum albumin; N, number of replicates; SD, standard deviation. ^a^ 10% FBS. ^b^ Calculated as CC_50_/EC_50_. ^c^ 10% FBS + 40% HS + 27 mg/mL of HSA. ^d^ Calculated as EC_50_ in high-serum conditions/EC_50_ in standard conditions.

**Table 2:** Estimated protein-binding-adjusted 90% effective concentration of VH3739937 against phenotyped HIV-1 strains.

Virus Name	HIV-1 Subtype	Gag Phenotype ^a^	EC_90_, nM	3× PBA-EC_90_, nM
NL_4-3_-P373S	B	Ref	6.8	299
1900140 ^b^	B	V362I	5.7	250
93BR022 ^b^	B	V370A	4.7	206
NL_4-3_-P373S/ΔV370	B	ΔV370	7.0	309
28-MDR ^b^	B	Many ^c^	8.0	354
11657-3 ^b^	C	R286K/ΔV370/ΔN375/M377L	10.0	456
Mean (SD), nM	—	—	—	312 (87)

EC_90_; 90% effective concentration; PBA-EC_90_, protein-binding-adjusted EC_90_; SD, standard deviation. ^a^ Relative to the NL_4-3_-P373S consensus sequence. ^b^ Recombinant RepRluc virus with *gag*/*pro* sequences derived from the listed clinical isolate. ^c^ HXB2 sequence.

**Table 3:** Antiviral activity of VH3739937 against HIV-1 and HIV-2 laboratory strains ^a^.

Laboratory Strain	Tropism	VH3739937 EC_50_, Mean (SD), nM	Atazanavir EC_50_, Mean (SD), nM
HIV-1			
NL_4-3_	CXCR4	3.1 (1.0)	1.9 (0.4)
HXB2	CXCR4	2.0 (1.6)	1.9 (0.7)
IIIB	CXCR4	1.6 (0.3)	2.6 (0.4)
LAI	CXCR4	1.3 (0.3)	1.3 (0.5)
MN	CXCR4	1.4 (0.4)	3.1 (1.7)
RF	CXCR4	4.4 (2.9)	1.6 (0.9)
BaL	CCR5	2.0 (0.5)	2.1 (1.0)
JRFL	CCR5	2.4 (2.0)	1.4 (1.5)
HIV-2			
ROD	CXCR4	1.3 (0.2)	2.0 (0.1)
287	CCR5/CXCR4	>750	36 (2.0)

EC_50_, half-maximal effective concentration; SD, standard deviation. ^a^ Average of 2 experiments, each performed in triplicate.

**Table 4:** Antiviral activity of VH3739937 against viruses containing Gag/protease sequences from clinical isolates on the NL_4-3_ background.

HIV-1 Subtype ^a^	Virus Name	EC_50_, nM
**Target Cell: PBMCs ^b^**		
A	I-2496	3.0
A	UG275	2.0
A	UG92031	2.0
CRF01_AE	42368	1.0
CRF01_AE	CM235	1.0
CRF01_AE	CM243	2.0
B	92HT593	1.0
B	92HT594	1.0
B	92HT596	2.0
B	92HT657	5.0
B	92US660	3.0
B	93US144	2.0
B	ASM57	2.0
B	ASM61	4.0
B	BR92030	3.0
B	BZ167	2.0
B	THA92014	5.0
C	97ZA009	5.0
C	ETH2220	5.0
C	I-2516	4.0
C	UG268	3.0
C	ZAM18	3.0
D	SE365	2.0
D	UG270	2.0
F	BZ126	2.0
**Target Cell: CEM-NKR-CCR5-Luc ^c^**		
A	93RW034	2.1
A	94UG103	1.5
CRF01_AE	92TH001	0.7
B	92BR003	0.20
B	92BR018	2.2
B	92BR028	2.7
B	93BR008	0.8
B	93BR017	1.5
B	93US143	1.7
C	20706-3	1.5
C	98BR004	1.6
C	MJ4	0.4
D	92UG046	2.0
D	94UG114	4.0
Type N	YBF30	3.1
Type O	BCF03	4.7

EC_50_, half-maximal effective concentration; PBMC, peripheral blood mononuclear cell. ^a^ Or type where indicated. ^b^ Evaluation of virus growth by reverse-transcriptase activity. ^c^ Evaluation of virus growth by the expression of a luciferase reporter in CEM-NKR-CCR5-Luc cells.

**Table 5:** Antiviral activity of VH3739937 against polymorphic viruses on an NLRepRlucP373S background.

Substitution ^a^	Multiple-Cycle Assay			Single-Cycle Assay		
	EC_50_, nM	FC-EC_50_	MPI, %	EC_50_, nM	FC-EC_50_	MPI, %
Wild type	2.0	Ref	99	5.0	Ref	99
V370A	4.0	2.0	98	6.0	1.2	99
ΔV370	4.0	2.0	98	12.0	2.4	100
V370A/ΔV371	5.0	2.5	92	ND	ND	ND
V362I/V370A	2.0	1.0	94	11.0	2.3	98
T332S/V362I/prR41G	8.0	4.0	98	24.0	4.8	96
A326T/V362I/V370A	4.0	2.0	95	ND	ND	ND
A364V	5.0	2.5	95	32.0	6.4	57
ΔV370/T371A	ND	ND	ND	7.0	1.4	99
A366V	ND	ND	ND	>3000	>600	−141 ^b^

EC_50_, half-maximal effective concentration; FC-EC_50_, half-maximal fold change effective concentration; MPI, maximal percent inhibition; ND, no data. ^a^ Known Gag polymorphisms identified in studies with bevirimat, GSK3532795, and/or GSK3640254. ^b^ A negative MPI indicates virus replication is enhanced compared with samples without VH3739937.

**Table 6:** Substitutions in Gag after dose-escalating resistance selection ^a^.

Virus	Gag Variants, % ^b,c^							
	D93N ^d^	H144Y	D298D/N	T332P	V362I	L363L/W	A364A/V ^e^	R384K ^f^
IIIB #1	-	-	-	-	-	20/80	-	-
IIIB #2	-	-	-	-	-	70/30	20/80	-
NL_4-3_ #1	100	-	-	100	-	-	-	-
NL_4-3_ #2	-	100	70/30	-	100	-	-	100

EC_50_, half-maximal effective concentration; PCR, polymerase chain reaction. ^a^ Final concentration of VH3739937 was 64× the EC_50_. ^b^ Substitutions are in the capsid protein unless otherwise noted. ^c^ Percentages estimated from sequence traces of PCR amplicons. ^d^ Located in the matrix protein. ^e^ Located in spacer peptide 1. ^f^ Located in the nucleocapsid protein.

**Table 7:** Susceptibility of NL_4-3_ viruses with substitutions identified during dose-escalating resistance selection to VH3739937.

Substitution	Multiple-Cycle Assay			Single-Cycle Assay		
	EC_50_, Mean (SD), nM	FC-EC_50_	MPI, %	EC_50_, Mean (SD), nM	FC-EC_50_	MPI, %
Wild type	2.8 (1.4)	Ref	93	2.8 (0.7)	Ref	98
D93N	3.3 (1.5)	1.2	95	5.7 (1.4)	2.1	98
H144Y	6.4 (3.9)	2.3	75 ^a^	11.8 (2.5)	4.2	94
V362I	6.8 (3.8)	2.5	93	ND	ND	ND
R384K	2.8 (1.5)	1.0	92	2.7 (0.9)	1.0	97
D298N	DNR			DNR		
T332P	DNR			>2000	>721	35
L363W	DNR			>2000	>721	37
D93N/T332P	DNR			>2000	>721	22
H144Y/V362I/R384K	DNR			42.0 (15)	15.0	78

DNR, did not replicate or produce signal; EC_50_, half-maximal effective concentration; FC-EC_50_, half-maximal fold change effective concentration; MPI, maximal percent inhibition; ND, no data. ^a^ Virus grew similarly poorly (MPI of ~74%) in the nelfinavir control sample.

## Data Availability

The data and study documents can be requested for further research from www.clinicalstudydatarequest.com.
